# Psychobiological benefits of Dhikrullah breathing for emotional and cognitive recovery

**DOI:** 10.1590/1980-5764-DN-2025-0454

**Published:** 2026-10-12

**Authors:** Dedy Syahrizal, Iskandar Iskandar, Subhan Rio Pamungkas, Agung Pranata, Rika Farhana, Nur Anisa Sarafina

**Affiliations:** 1 Universitas Syiah Kuala, Faculty of Medicine, Department of Biochemistry – Banda Aceh, Aceh, Indonesia.; 2 Universitas Syiah Kuala, Faculty of Medicine, Psychology Program – Banda Aceh, Aceh, Indonesia.; 3 Universitas Syiah Kuala, Faculty of Medicine, Department of Mental Health Sciences – Banda Aceh, Aceh, Indonesia.; 4 Universitas Syiah Kuala, Faculty of Medicine, Department of Parasitology – Banda Aceh, Aceh, Indonesia.; 5 Universitas Syiah Kuala, Faculty of Medicine, Master Program of Biomedical Science – Banda Aceh, Aceh, Indonesia.; 6 Universitas Syiah Kuala, Faculty of Medicine, Medical Program – Banda Aceh, Aceh, Indonesia.

**Keywords:** Anxiety, Breathing Exercises, Hydrocortisone, Memory, Spiritual Therapies, Ansiedade, Exercícios Respiratórios, Hidrocortisona, Memória, Terapias Espirituais

## Abstract

Long-term anxiety disrupts emotional, hormonal, and cognitive systems, including elevated cortisol and impaired memory performance.

**Objective:**

This quasi-experimental study evaluated the effects of Dhikrullah breathing therapy on anxiety symptoms, serum cortisol, brain-derived neurotrophic factor (BDNF), and memory performance.

**Methods:**

Anxiety levels were assessed using the Depression Anxiety Stress Scales (DASS-42), cortisol and BDNF concentrations were measured using an enzyme-linked immunosorbent assay (ELISA), and verbal memory was evaluated using a standardized word recall task.

**Results:**

Statistical analyses using the Wilcoxon signed-rank test and the paired t-test revealed significant reductions in anxiety (p=0.001) and cortisol levels (p=0.001), as well as a significant improvement in memory performance (p=0.001). In contrast, serum BDNF levels showed a small numerical decrease that was not statistically significant (p=0.284). Correlational analysis showed a moderate positive correlation between changes in BDNF and memory performance (r=0.54; p=0.01), as well as a moderate negative correlation between changes in BDNF and cortisol (r=–0.51; p=0.04). In addition, changes in anxiety were moderately negatively correlated with changes in memory performance (r=–0.50; p=0.005), whereas the correlation between changes in anxiety and cortisol was weak and not statistically significant (r=0.39; p=0.17).

**Conclusion:**

Dhikrullah breathing therapy promotes positive changes in the emotional, hormonal, and cognitive responses of people who experience anxiety. Additional controlled studies are needed to determine the potential use of this treatment in both clinical and community mental health settings.

## RESUMO

A ansiedade de longo prazo perturba os sistemas emocional, hormonal e cognitivo, incluindo níveis elevados de cortisol e comprometimento do desempenho da memória.

**Objetivo:**

Este estudo quase-experimental avaliou os efeitos da terapia respiratória Dhikrullah sobre os sintomas de ansiedade, o cortisol sérico, o fator neurotrófico derivado do cérebro (BDNF) e o desempenho da memória.

**Métodos:**

Os níveis de ansiedade foram avaliados por meio da Escala de Depressão, Ansiedade e Estresse-42 (DASS-42); as concentrações de cortisol e BDNF foram medidas por ensaio de imunoabsorção enzimática (ELISA); e a memória verbal foi avaliada por meio de uma tarefa padronizada de recordação de palavras.

**Resultados:**

Análises estatísticas utilizando o teste de Wilcoxon de postos sinalizados e o teste t para amostras emparelhadas revelaram reduções significativas na ansiedade (p=0,001) e nos níveis de cortisol (p=0,001), bem como uma melhora significativa no desempenho da memória (p=0,001). Em contrapartida, os níveis séricos de BDNF apresentaram uma pequena redução numérica que não foi estatisticamente significativa (p=0,284). A análise correlacional revelou uma correlação positiva moderada entre as variações no BDNF e o desempenho da memória (r=0,54; p=0,01), bem como uma correlação negativa moderada entre as variações no BDNF e o cortisol (r=–0,51; p=0,04). Além disso, as variações na ansiedade apresentaram correlação negativa moderada com as variações no desempenho da memória (r=–0,50; p=0,005), enquanto a correlação entre as variações na ansiedade e o cortisol foi fraca e não estatisticamente significativa (r=0,39; p=0,17).

**Conclusão:**

A terapia respiratória Dhikrullah promove mudanças positivas nas respostas emocionais, hormonais e cognitivas de pessoas que apresentam ansiedade. São necessários estudos controlados adicionais para determinar o potencial de uso desse tratamento tanto em ambientes clínicos quanto comunitários de saúde mental.

## INTRODUCTION

Anxiety disorders are among the most prevalent psychiatric conditions worldwide, exerting substantial impacts on cognitive function, neuroendocrine regulation, and overall quality of life. Recent global estimates indicate that over 301 million individuals, or approximately 4.05% of the population, are affected by anxiety-related conditions — a figure that has increased by 55% over the past three decades, particularly in high-income countries^1^. In Indonesia, national health surveys report that 6% of individuals aged 15 and above, equating to roughly 14 million people, exhibit anxiety symptoms^2^, suggesting a significant public health burden within a culturally unique population.

At the neurobiological level, the pathophysiology of anxiety involves dysregulation of the hypothalamic–pituitary–adrenal (HPA) axis. Prolonged exposure to psychosocial stressors activates this axis, resulting in hypersecretion of adrenocorticotropic hormone and elevated circulating cortisol levels^3^. Cortisol, a glucocorticoid essential for metabolic homeostasis and immune modulation, becomes maladaptive under chronic activation, contributing to hippocampal atrophy, impaired memory, and emotional instability^4^.

Although pharmacological strategies remain the mainstay of anxiety treatment, non-pharmacological interventions have gained traction for their integrative and patient-centered approach^5^. Among these, spiritual and mindfulness-based therapies offer unique psychosomatic benefits by modulating stress response pathways. Dhikr, an Islamic contemplative practice characterized by rhythmic verbal repetition and breath synchronization, is proposed to influence the HPA axis via parasympathetic activation and neuroendocrine recalibration. Preliminary evidence indicates that Dhikrullah-based breathing interventions can lower cortisol levels across diverse populations, including postmenopausal women, individuals in drug rehabilitation, and patients with chronic stress^6,7^.

Yet, the psychoneurobiological underpinnings of Dhikr remain poorly elucidated, particularly regarding its effects on cognitive biomarkers such as brain-derived neurotrophic factor (BDNF) — a key regulator of synaptic plasticity and memory consolidation. To date, no study has comprehensively assessed the integrated effects of Dhikrullah breathing therapy on neuroendocrine (cortisol), affective (anxiety), and cognitive (BDNF and memory) domains using objective biological markers.

In this study, the multidimensional impact of Dhikrullah breathing therapy on individuals with anxiety was exemined, incorporating physiological, cognitive, and psychological endpoints. It was hypothesized that the intervention would significantly reduce anxiety symptoms and cortisol levels, while concurrently enhancing BDNF expression and verbal memory performance, thereby advancing the neurobiological understanding of spiritually integrated therapies.

## METHODS

### Study design and ethical considerations

This study employed a quasi-experimental, one-group pretest–posttest design to evaluate the physiological and psychological effects of Dhikrullah breathing therapy. The research was conducted between September and November 2023 in Syiah Kuala District, Banda Aceh, Indonesia. Ethical clearance was obtained from the Medical Research Ethics Committee of the Faculty of Medicine, Syiah Kuala University (Reference No. 179/EA/FK/2023). All procedures adhered to the ethical standards of the Declaration of Helsinki. Each participant provided written informed consent prior to participation.

### Participants and sampling procedure

The research used a quasi-experimental pretest-posttest design. Participants were selected using purposive sampling through community outreach and referral from local health posts. Inclusion criteria were as follows: being Muslim, aged between 18 and 30 years, residing in Syiah Kuala District, and experiencing symptoms of anxiety within the previous two weeks as determined during initial screening. Participants were excluded if they were currently using anxiolytics, antidepressants, or antipsychotics; had a history of severe respiratory illness; or were unable to complete the full intervention protocol. Individuals who missed intervention sessions or withdrew consent were also excluded. A total of 20 eligible participants were enrolled, and their demographic information was collected at baseline. The sample size was determined using the Levy and Lemeshow^8^ formula, as shown in Equation 1:


(1) $$n=\frac{2\sigma^{2}{\left(z_{1-\alpha }+z_{1-\beta }\right)}^{2}}{{\left(\mu_{1}-\mu_{2}\right)}^{2}}$$

Where:

*n*: standard deviation (4.04);

*Z*_1—α_: 1.96 (95% confidence level);

*Z*_1—β_: 1.28 (90% statistical power);

μ_1_ – μ_2_: expected mean difference (4.81).

Based on these parameters, the minimum sample size required was 15 participants. To account for potential attrition, loss to follow-up, or non-adherence, a 10% correction factor was applied, as shown in Equation 2:


(2) $$n=\frac{15}{1-0.1}=17$$

The final sample size was rounded to 20 participants to ensure sufficient statistical power.

### Anxiety assessment instrument

Anxiety symptoms were assessed using the validated Indonesian version of the Depression, Anxiety Stress Scales (DASS-42), developed by Lovibond and Lovibond^9^. The anxiety subscale consists of 14 items evaluating physiological arousal and cognitive anxiety. Each item is rated on a 4-point Likert scale, ranging from 0 (“Did not apply to me at all”) to 3 (“Applied to me very much or most of the time”). The sum of the scores yields a total anxiety score that is categorized as normal (0–7), mild (8–9), moderate (10–14), severe (15–19), or extremely severe (≥20). Participants completed the DASS-42 questionnaire both before and after the intervention. Participants were eligible for inclusion if their anxiety score was ≥8 (mild to extremely severe). Those with scores in the normal range of 0–7 were not included. Most individuals had moderate anxiety (40%) at baseline, followed by mild anxiety (35%), severe anxiety (15%), and extremely severe anxiety (10%).

### Collection and handling of biological samples

All participants had venous blood samples (5 mL each) drawn twice: once before the Dhikrullah breathing intervention (baseline measurement) and again on the seventh day after the intervention ended. The blood samples were taken between 8 and 10 AM, which is when endogenous cortisol secretion peaks, so order to account for the circadian variability of cortisol. Blood was drawn into serum separator tubes, allowed to clot for two hours at room temperature or overnight at 4°C, and then centrifuged at 1000×g for 20 minutes. The serum was separated into aliquots and stored at –20°C until analysis. All procedures were conducted under aseptic conditions to avoid contamination and preserve sample integrity. To provide temporal standardization, samples were taken both before and after the intervention over the same time period. Sample collection was conducted by trained laboratory personnel at the Syiah Kuala University Teaching Hospital.

### Measurement of cortisol and brain-derived neurotrophic factor using enzyme-linked immunosorbent assay

Serum cortisol and BDNF concentrations were quantified using human-specific ELISA kits (ELK Biotechnology, Wuhan, China)^10^. For cortisol, the Human Cortisol ELISA Kit (Cat. No. ELK8526) was used, and for BDNF, the Human BDNF ELISA Kit was employed. Prior to the assay, all reagents and serum samples were brought to room temperature. A total of 50 μL of standard solution or sample was pipetted into designated wells, followed by the addition of 50 μL of biotinylated antibody solution. The plate was incubated at 30°C for 60 minutes. After washing three times with 1× buffer, 100 μL of streptavidin-HRP was added, and the plate was incubated again at 37°C for 50 minutes. Plates were then washed five times, and 100 μL of TMB substrate was added to each well, followed by a 20-minute incubation. Finally, 50 μL of stop solution was added, and absorbance was read immediately at 450 nm using a microplate reader. A standard curve was generated for each biomarker, and serum concentrations were calculated according to the manufacturer’s instructions.

### Dhikrullah breathing therapy intervention

The intervention was delivered by a certified Islamic trainer experienced in conducting spiritual breathing therapy. All sessions were held in a quiet, spacious room with natural lighting and minimal external distractions. Before each session, participants performed ritual ablution (wudhu) to enhance mindfulness and spiritual readiness. Participants underwent ceremonial cleansing (wudhu) before every session as part of the regular spiritual preparation for Dhikr practice. The goal of this phase was to improve spiritual engagement and attentional focus. The possible independent psychophysiological effects of ablution, such as sensory stimulation from exposure to water, autonomic modulation, relaxation responses, or increased focused attention, were not independently examined or controlled. Therefore, when analyzing the standalone effects of the intervention, ablution might be a potential confounding factor. The Dhikrullah breathing intervention used deep diaphragmatic breathing, which followed a controlled breathing rate of 15–20 breaths per minute. The breathing pattern began with a 1–2-second nasal inhalation and ended with a 2-second oral exhalation during which the word “Allah” was softly vocalized in synchrony with the breath. The session lasted three minutes as participants remained seated in a silent environment with minimal external disruptions. The research team monitored all participants to check their breathing speed and method while they watched for any potential side effects.

After the first supervised session, participants continued practicing independently five times per day for seven consecutive days. Daily practice was recorded on individual checklist sheets, and adherence was reinforced by daily reminders via text messages and phone calls from the research team.

### Statistical analysis

All statistical analyses were performed using MATLAB R2024a (MathWorks Inc., Natick, MA, USA). Descriptive statistics, including means, standard deviations, medians, and interquartile ranges, were computed to summarize participant demographics and baseline psychobiological measures. To assess the normality of continuous data distributions (e.g., anxiety scores, cortisol, BDNF, and memory), the Shapiro–Wilk test was conducted using the swtest function from MATLAB’s File Exchange or custom statistical scripts. For outcome variables exhibiting non-normal distributions (cortisol levels, anxiety scores, BDNF levels, and memory scores), Wilcoxon signed-rank tests were employed to compare pre– and post-intervention values, implemented using MATLAB’s signrank function. Effect sizes were calculated using the rank-based effect size (r). They were interpreted as small (r≥0.10), moderate (r≥0.30), and large (r≥0.50), and were computed using MATLAB scripts based on Equation 3:


(3) $$r=\frac{Z}{\sqrt{N}}$$

Where:

Z: is the standardized test statistic obtained from the Wilcoxon signed-rank test; and

N: is the number of paired observations included in the analysis.

To investigate interrelationships among outcome variables, Spearman’s rho correlation coefficients were computed using the corr function with the parameters ‘Type’, ‘Spearman’ to evaluate associations between change scores (Δ) in anxiety, cortisol, BDNF, and memory performance. Visualizations including correlation heatmaps and bar charts of effect sizes were generated using MATLAB’s heatmap and bar functions, respectively. Statistical significance was defined as p<0.05 for all analyses. All scripts were validated using test datasets and cross-verified with manual calculations to ensure computational accuracy.

## RESULTS

### Participant characteristics and baseline profiles

All 20 participants enrolled in the study completed the seven-day Dhikrullah breathing therapy intervention and follow-up assessments, resulting in a 100% retention rate. The average participant age was 21.2 years, ranging from 18 to 30 years. The majority of participants were female (80%; n=16), with 20% being male (n=4). Most participants were aged 18–25 years (90%; n=18), while 10% (n=2) were aged 26–30 years. The study included participants who were both Muslim and unmarried because these criteria matched the religious and spiritual requirements of the Dhikrullah breathing intervention. The study sample included 90% of participants who were undergraduate medical students, while 10% of participants were licensed general practitioners. Most participants were Acehnese (85%), while the remaining were from Mandailing, Minang, and Malay ethnic groups, indicating limited ethnic diversity within the sample.

Potential adverse effects, such as lightheadedness, signs of hyperventilation, respiratory discomfort, or psychological distress, were tracked in the participants. Neither during nor after the intervention period were any adverse effects noted. Thus, none of the participants discontinued the intervention, resulting in a 0% adverse event rate.

At baseline, participants exhibited elevated levels of psychological distress and physiological arousal. The mean DASS-42 anxiety score was 13.85 (standard deviation [SD] ±6.09; median: 15.0; 95% confidence interval [CI] 10.99–16.71), positioning most individuals in the “severe” clinical category. The mean serum cortisol concentration, indicative of HPA axis activity, was 8.69 μg.dL^-1^ (SD±3.60; median: 8.25; 95%CI 7.00–10.38), suggesting elevated physiological stress levels with reasonable precision of estimation. Serum levels of BDNF were relatively low (mean: 478.04 pg.mL^-1^; SD±159.70; median: 506.0; 95%CI 403.29–552.79), suggesting limited neuroplastic support. Cognitive performance, measured through a verbal memory task, was moderate at baseline (mean: 12.65 words; SD±1.99; median: 13.0; 95%CI 11.72–13.58), reflecting relatively stable baseline memory performance within the sample^11^ (Table 1).

**Table 1.** Summary of outcome measures pre- and post-intervention.

Variable	Pretest (mean±SD)	Posttest (mean±SD)	Pretest (median)	Posttest (median)	Z-score	p-value	Significance
DASS-42 (Anxiety)	13.85±6.09	5.45±4.74	15.0	4.5	–3.92	0.001	Highly significant
Memory (total words)	12.65±1.99	13.75±1.62	13.0	14.0	–3.86	0.001	Highly significant
BDNF (pg.mL^-1^)	478.04±159.70	453.34±145.58	506.0	480.0	–1.07	0.284	Not significant
Cortisol (μg.dL^-1^)	8.69±3.60	5.60±2.36	8.25	5.50	–3.38	0.001	Highly significant

Abbreviations: DASS, Depression Anxiety Stress Scales; BDNF, brain-derived neurotrophic factor.

Following the intervention, participants demonstrated statistically and clinically significant improvements across psychological, endocrine, and cognitive domains. After the intervention, 15 participants (75%) shifted into the “normal” anxiety category (DASS-42 score ≤7), indicating clinically meaningful symptom reduction across severity levels. The mean DASS-42 anxiety score dropped to 5.45 (SD±4.74; median: 4.5; 95%CI 3.23–7.67), representing a 60.65% reduction. This change was highly significant (Wilcoxon Z=–3.92; p=0.001) and corresponded to a categorical shift in anxiety severity based on established DASS-42 cut-off thresholds, with several participants moving from the “severe” or “moderate” ranges to “mild” or “normal” classifications. The relatively narrow post-intervention confidence interval indicates acceptable precision of estimation despite the modest sample size, further supporting the robustness of the observed clinical improvement. In, serum cortisol levels declined to 5.60 μg/dL (SD ±2.36; median: 5.50), a 35.6% reduction (Z=–3.38; p=0.001), indicating substantial HPA axis downregulation and reduced physiological stress. Serum BDNF levels showed a modest numerical decrease rather than the anticipated rise (mean: 453.34 pg.mL^-1^; SD ±145.58; median: 480.0), and this change was not statistically significant (Z=–1.07; p=0.284). Memory performance improved significantly following the intervention. The mean score increased from 12.65 (SD±1.99; 95%CI 11.77–13.33) at baseline to 13.75 (SD±1.62; 95%CI 13.07–14.73) post-intervention. These findings show significant pre–post differences in anxiety, cortisol, and memory performance, whereas no significant group-level change was observed in BDNF. However, because of the one-group pre–post design, these results should be interpreted as preliminary and do not permit causal inference regarding the effects of the intervention.

### Pre–post intervention outcomes

The visual representation in Figure 1 illustrates both pre- and post-intervention results, which demonstrate the impact of the intervention on all main psychobiological research variables. The DASS-42 anxiety scores in Figure 1A show a major decrease, as the mean score dropped from 13.85 before the intervention to 5.45 after the seven-day Dhikrullah breathing practice. The individual trajectories of all participants moved downward, indicating that they all experienced emotional progress. The verbal memory performance shown in Figure 1B improved when participants increased their mean word recall scores from 12.65 to 13.75. Most participants presented better cognitive function abilities because they maintained better stress levels and improved their ability to concentrate. Figure 1C shows a small decrease in serum BDNF levels from pre- to post-intervention. While some participants exhibited higher values, the results were highly heterogeneous, and the mean change was not statistically significant. Figure 1D shows a decrease in serum cortisol levels from 8.69 μg/dL at baseline to 5.60 μg.dL^-1^ post-intervention, with most participants demonstrating lower values. The findings indicate that Dhikr breathing functions as a method to control HPA axis activity and decrease physiological stress levels. The visual displays demonstrate the average results of the study together with the different outcomes experienced by each participant, thereby increasing the study’s transparency and illustrating the strength of the intervention outcomes.

**Figure 1.**
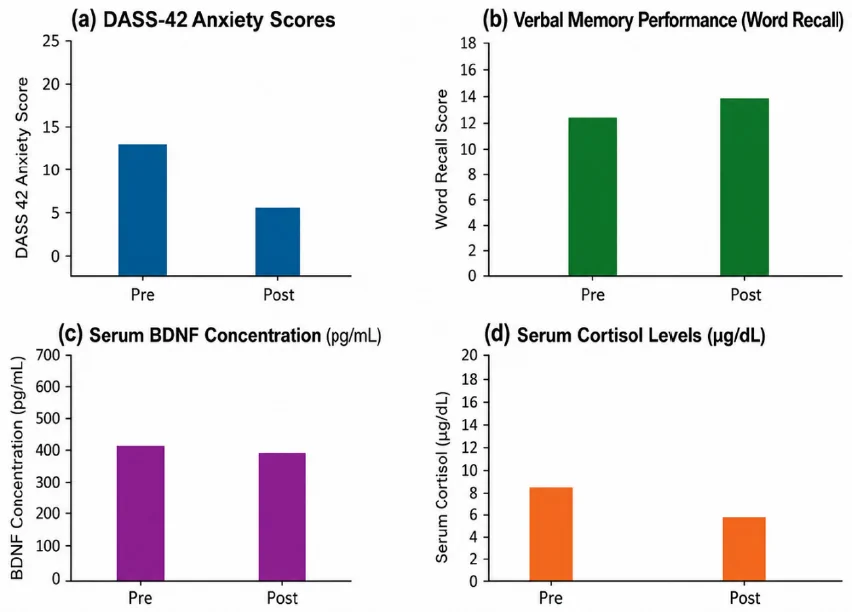
Pre-and post-intervention scores across psychobiological domains. (A) DASS-42 Anxiety Scores; (B) Verbal Memory Performance (Word Recall); (C) Serum BDNF concentration (pg.mL^-1^); and (D) serum cortisol levels (μg.dL^-1^).

DASS, Depression Anxiety Stress Scales; BDNF, brain-derived neurotrophic factor.

Figure 2 shows anxiety scores measured before the intervention and after it had been completed. The figure depicts a marked reduction in DASS-42 anxiety scores, with the mean score decreasing from 13.85 at baseline to 5.45 after seven days of Dhikrullah breathing therapy.

**Figure 2.**
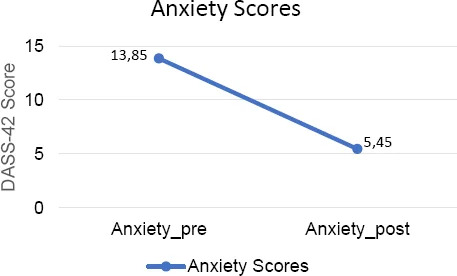
Pre- and post-intervention anxiety scores following Dhikrullah breathing therapy.

### Interrelationships between psychobiological outcomes

To investigate the interconnections among emotional, neuroendocrine, and cognitive adaptations induced by the intervention, correlational analyses were conducted using change scores (Δ) for each outcome. Figure 3 presents the Spearman correlation matrix for changes in anxiety, cortisol, BDNF, and memory performance. The results revealed several meaningful associations (Table 2). A moderate positive correlation was observed between changes in BDNF and changes in memory performance (r=0.54; p=0.01), suggesting that enhanced neuroplasticity may support cognitive recovery. In addition, changes in BDNF were moderately negatively correlated with changes in cortisol (r=–0.51; p=0.04), indicating that neuroendocrine recovery may be linked with neurotrophic regulation. Notably, reductions in anxiety scores were moderately negatively correlated with memory improvement (r=–0.50; p=0.005), implying that emotional stabilization may contribute to improved cognitive processing. The correlation between changes in anxiety and cortisol was weak and not statistically significant (r=0.39; p=0.17), suggesting that psychological and physiological responses to the intervention may be mediated through partially distinct mechanisms. These findings suggest that although mean BDNF levels did not change significantly at the group level, individual variations in BDNF were associated with cognitive and endocrine responses associated with the intervention.

**Figure 3.**
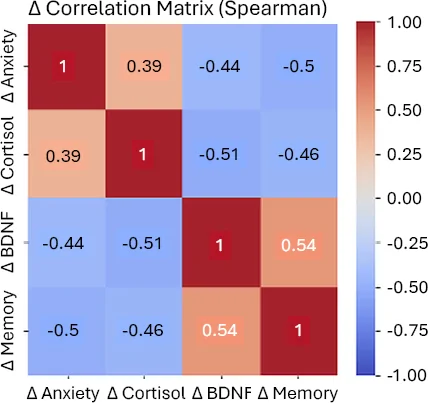
Psychobiological effects of Dhikrullah breathing therapy on Spearman correlation.

**Table 2.** Correlation matrix of changes in key variables.

Variable pair	Correlation (r)	p-value	Interpretation
ΔBDNF *vs* ΔMemory	0.54	0.010	Moderate positive
ΔBDNF *vs* ΔCortisol	-0.51	0.040	Moderate negative
ΔAnxiety *vs* ΔMemory	-0.50	0.005	Moderate negative
ΔAnxiety *vs* ΔCortisol	0.39	0.170	Weak, not significant

## DISCUSSION

### Anxiety reduction and emotional stabilization

The remarkable decline in anxiety levels observed following the Dhikrullah breathing intervention reflects not only statistical significance but also a clinically meaningful transformation in emotional well-being, characterized by a mean DASS-42 anxiety score reduction from 13.85 to 5.45, marking a 60.65% decrease. This magnitude of change, confirmed by the Wilcoxon signed-rank test (Z=–3.92; p=0.001), situates the post-intervention cohort within the “normal to mild” anxiety spectrum, a dramatic departure from the pre-intervention “severe” classification. Such findings suggest the potential relevance of culturally familiar, spiritually guided breathing techniques in non-pharmacological mental health interventions; however, the present design does not allow conclusions about therapeutic efficacy (Figure 2)^12^. This pattern of improvement in anxiety symptoms was consistently observed throughout the study. The rhythmic repetition of divine phrases in Dhikrullah — synchronized with slow, diaphragmatic breathing — is postulated to activate the parasympathetic nervous system while simultaneously dampening sympathetic arousal, thereby rebalancing autonomic dysregulation often found in anxiety disorders^13^. Additionally, this practice likely engages cortical-limbic networks involved in emotional processing, including the anterior cingulate cortex and medial prefrontal cortex, regions implicated in mindfulness and affective control^14^. The observed improvement in emotional symptoms over seven consecutive days suggests that Dhikrullah breathing may have potential as an adjunctive practice for anxiety management, although this interpretation remains preliminary and requires confirmation in controlled studies. Moreover, the universal acceptability of this approach among Muslim participants, due to its alignment with religious beliefs, enhances its feasibility and sustainability within community-based mental health programs.

The Spearman correlation matrix displays results from the study, showing changes in anxiety levels, cortisol, BDNF, and memory performance after participants completed seven days of Dhikrullah breathing exercises. The heatmap displays the intensity of associations among psychobiological variables, with red tones indicating positive correlations and blue tones indicating negative correlations (Figure 3).

The research found that changes in BDNF and memory performance showed a moderate positive correlation (r=0.54; p=0.01). This association supports the theoretical link between neurotrophic regulation and cognitive adaptation. The research found that changes in BDNF and cortisol levels showed a moderate negative correlation (r=–0.51; p=0.04). Participants who experienced cortisol reductions tended to exhibit better neurotrophic dynamics, suggesting that their stress hormone downregulation interacted with their neurobiological recovery processes. In addition, the matrix displayed a moderate negative correlation between anxiety reduction and memory improvement (r=–0.50; p=0.005). The moderate negative correlation indicates that greater emotional stabilization was closely associated with enhanced cognitive performance. The finding aligns with neurocognitive models, which propose that reduced affective distress may restore attentional and hippocampal functioning. Finally, the correlation between changes in anxiety and cortisol was weaker and not statistically significant (r=0.39; p=0.17), indicating that psychological and endocrine adaptations may not occur in strict parallel and could be mediated by partially distinct regulatory pathways. Overall, the correlation matrix highlights the interconnected nature of emotional, endocrine, and cognitive adaptations following Dhikrullah breathing therapy. Rather than functioning as isolated outcomes, these domains appeared dynamically coupled, supporting a multidimensional psychobiological model of intervention response.

### Correlational dynamics between psychobiological variables

The observed interrelationships among changes in anxiety, cortisol, BDNF, and memory performance suggest a possible psychobiological pattern in which cognitive, emotional, and endocrine functions may be related. However, these correlational findings should be interpreted cautiously and do not establish a therapeutic mechanism. A moderate negative correlation between anxiety reduction and memory improvement (r=–0.50) highlights a well-documented yet often underexplored phenomenon: the alleviation of affective symptoms can lead to measurable cognitive gains, particularly in attention, encoding, and retrieval processes^15^. This finding resonates with theoretical frameworks in neuropsychology suggesting that chronic anxiety consumes prefrontal attentional resources and disrupts hippocampal functioning, thus impairing working memory; conversely, emotional relief can restore neural efficiency across these domains^16^. The positive correlation between changes in BDNF and memory performance suggests a possible association between neurotrophic dynamics and cognitive adaptation. BDNF results should be considered exploratory data that may generate hypotheses for future research, as no significant mean-level change in BDNF was observed^17^. Furthermore, the moderate negative correlation between changes in BDNF and cortisol (r=–0.51; p=0.04) suggests a possible association between neurotrophic and endocrine responses. However, this finding does not demonstrate that Dhikrullah breathing directly modulates the HPA axis or promotes trophic factor expression^18^. Interestingly, the correlation between cortisol and anxiety changes (r=0.39) was weaker and not statistically significant, suggesting that psychological improvements may precede, or follow a different trajectory from, endocrine recalibration. This dissociation emphasizes the importance of evaluating multiple biomarkers in psychotherapeutic trials, as relying solely on self-reported affective symptoms may obscure significant physiological changes. Collectively, these correlational findings suggest that emotional, hormonal, and neurocognitive changes may occur in parallel; however, the present study cannot determine whether these patterns reflect direct effects of the intervention or other uncontrolled factors.

### Effect size estimation, biological magnitude, and holistic interpretation

The results of effect size calculations used to assess primary outcomes proved that the findings reached full statistical strength while showing practical applicability and biological importance. Memory improvement following the intervention showed an effect size of approximately r=0.86, which exceeded the established large effect threshold of r≥0.50. The study found that anxiety and cortisol levels decreased significantly, while serum BDNF levels showed no statistically significant difference between the two measurement points. The findings revealed that BDNF changes correlated with memory improvement and cortisol reduction, but the study did not produce significant neurotrophic growth because BDNF levels did not reach a substantial average increase. The research results regarding BDNF should be interpreted with caution given that they do not demonstrate direct neuroplastic development. The observed correlations suggest that individual variations in BDNF dynamics may be associated with cognitive and endocrine responses; however, correlational relationships do not imply causation. The existing associations between these two factors probably arise from shared control systems that do not directly impact neurotrophic activity.

Moreover, the use of culturally relevant spiritual practices amplifies the intervention’s accessibility and ecological validity. In Muslim-majority populations, Dhikrullah is not an alien or intrusive protocol but rather a respected and familiar practice imbued with intrinsic motivational and spiritual value^19^. This inherent congruence between belief and behavior may enhance placebo-independent engagement and adherence, which are critical factors for the long-term sustainability of mental health interventions. When viewed through holistic lens, Dhikrullah breathing therapy may be conceptualized as a culturally grounded biopsychosocial practice that is potentially relevant to neuroendocrine, psychological, and cognitive domains^20^. However, the present design does not allow firm conclusions regarding its specific mechanisms of action. As such, its utility may extend far beyond acute symptom relief, offering a model for integrative, community-based, and spiritually coherent mental health care.

The current research results require cautious interpretation because the study has both methodological and contextual limitations. The study used a single-arm pre–post design because it lacked a control group, which restricts all causal conclusions. The study found statistically significant changes, but the results do not prove that the intervention caused these changes. The absence of a comparison group creates a risk that placebo effects, regression to the mean, expectancy bias, and other nonspecific therapeutic effects commonly observed in behavioral intervention studies may have influenced the findings.

The study findings have two limitations related to the study design and the specific sociocultural context of the research sample. Dhikrullah breathing combines religious recitation with controlled breathing techniques, which depend on participant belief systems to determine their engagement, expectancy, and treatment outcomes. The research sample contained only Muslim participants from one geographic area, which means that the psychobiological findings cannot be used to make predictions about non-Muslim groups or secular settings until further empirical studies are conducted. It remains unclear to what extent the observed effects derive from spiritual meaning, religious congruence, or the physiological mechanisms of slow breathing itself.

The research requires attention to the essential methodological requirement that all participants must perform wudhu ritual cleansing before they start their breathing exercises. Multiple pathways exist which enable ablution to produce psychobiological effects according to different possible mechanisms. The body enters a state of relaxation through cool water exposure, which activates parasympathetic pathways together with tactile stimulation that promotes autonomic system balance. The ritualistic aspect of ablution enhances participants’ ability to concentrate their attention because it establishes expectations while it creates a spiritual state that helps them do the upcoming breathing task. The combination of sensory, cognitive, and spiritual functions resulted in significant reductions in anxiety and cortisol levels, which also led to better cognitive focus and memory performance. The study intervention should be viewed as a complete spiritual behavior program that includes multiple elements beyond the breathing practice. The present research design prevents determination of the extent to which ablution practice affects respiratory control. The research should include this component by using factorial or randomized control designs for future studies. The comparison groups should consist of:

- Dhikr breathing without prior ablution;
- ablution without structured breathing;
- secularized slow breathing protocols; or
- sham/waitlist controls.

These methodological improvements will help identify spiritual priming effects and physiological effects of breathing, thereby boosting the ability to draw causal connections and understand mechanisms.

Future research should conduct randomized controlled studies or sham-controlled studies that include comparison groups involving non-spiritual slow breathing, guided relaxation, and waitlist controls across various religious and cultural groups. The research methods will determine which phenomena are specific to certain contexts while demonstrating which others occur in all settings.

Furthermore, using more exacting experimental designs, future research should specifically account for the possible impacts of ritual cleansing by including comparison conditions such as:

- Dhikr breathing without preceding ablution;
- ablution without structured breathing; or
- secularized slow-breathing procedures that remove spiritual preparation, which could be included in factorial or randomized controlled experiments.

By disentangling the separate and combined effects of breathing and ablution components, these methodological improvements would strengthen causal inference and shed light on the mechanisms behind the reported psychobiological consequences.

### Strengths and limitations

This study presents several notable strengths. The pre–post quasi-experimental design created a framework that assessed multiple dimensions of the Dhikrullah breathing intervention through three distinct psychological, neuroendocrine, and cognitive assessment methods. The combination of DASS-42 and standardized verbal memory tasks with serum cortisol and BDNF biological indicators established strong construct validity while making research methods more reliable. The research used an established intervention method that was implemented in a controlled setting while participant compliance was monitored to produce reliable results. The results gained extra visibility through individual-level pre–post trajectory visualization, which boosted both visibility and reproducibility of the research results. The research team determined the sample size through statistical power calculation, which ensured that recruitment followed established scientific methods instead of being done for arbitrary reasons.

This study contains multiple limitations that need to be recognized. It included 20 participants because the sample size was determined based on statistical requirements. However, the small sample size made the results less applicable to other populations and limited their statistical representativeness. The study population was restricted to Muslim participants from a single geographic region and was predominantly composed of young female medical students. The study found that the specific demographic group that participated in the research created a major restriction because it prevented researchers from applying the results to non-Muslim, male, older adult, or ethnically different populations.

The research used a one-group pre–post quasi-experimental design, which lacked a control group, so it could not establish cause-and-effect relationships between variables. The significant improvements occurred because participants experienced expectancy effects, which caused their results to return to normal levels while they received placebo treatments and experienced other nonspecific therapeutic effects that researchers observed in behavioral research studies. Conclusions about persistent or long-term neurobiological adaptation are also precluded by the brief intervention duration. Furthermore, there was insufficient control over possible hormonal and diurnal variations that could affect BDNF and cortisol levels.

The use of serum BDNF as a proxy marker for central neuroplasticity should be approached with careful consideration. Peripheral BDNF levels may not directly reflect brain-derived BDNF activity due to differences in blood–brain barrier dynamics, platelet storage, and systemic influences. The interpretation of serum BDNF as an indicator of neural plasticity, therefore, remains conceptually limited, particularly in short-duration behavioral interventions.

Future studies should use multi-site, randomized controlled designs with active or sham comparator groups, longer follow-up periods, and larger, more religiously diverse samples. The uniqueness, long-term effects, and generalizability of psychobiological therapies based on Dhikrullah would be clarified by such methods.

In conclusion, this study provides preliminary evidence of pre–post improvements in anxiety, cortisol, and memory performance among individuals who participated in Dhikrullah breathing therapy. However, because this was a one-group pre–post study, these findings should be interpreted as associative and exploratory rather than causal. Within just seven days, participants exhibited significant reductions in anxiety symptoms and serum cortisol levels, alongside improved memory performance. In contrast, serum BDNF levels showed a small numerical decrease that was not statistically significant. These findings suggest that Dhikrullah breathing may be associated with changes in stress-related biological and cognitive outcomes, but the present study does not permit mechanistic conclusions.

Correlational analyses further suggest that emotional, endocrine, and cognitive adaptations may be interrelated. Although mean BDNF levels did not change significantly, changes in BDNF were positively associated with memory gains and inversely associated with cortisol changes, while reductions in anxiety were related to cognitive improvement. This integrated pattern suggests that spiritually informed mind–body interventions may be relevant to multiple dimensions of human functioning, although this possibility should be confirmed in controlled studies.

Given its accessibility and cultural congruence, Dhikrullah breathing therapy may warrant further investigation as a non-pharmacological approach in mental health care, particularly in Muslim-majority or resource-limited contexts. Future studies employing larger, randomized controlled trials with long-term follow-up and neuroimaging assessments are warranted to confirm these findings and further elucidate the neural mechanisms of change.

## Data Availability

The datasets generated and/or analyzed during the current study are available from the corresponding author upon reasonable request.
